# Case Report: Embedding “Digital Chronotherapy” Into Medical Devices—A Canine Validation for Controlling Status Epilepticus Through Multi-Scale Rhythmic Brain Stimulation

**DOI:** 10.3389/fnins.2021.734265

**Published:** 2021-09-24

**Authors:** Mayela Zamora, Sebastian Meller, Filip Kajin, James J. Sermon, Robert Toth, Moaad Benjaber, Derk-Jan Dijk, Rafal Bogacz, Gregory A. Worrell, Antonio Valentin, Benoit Duchet, Holger A. Volk, Timothy Denison

**Affiliations:** ^1^Institute of Biomedical Engineering, Department of Engineering Science, University of Oxford, Oxford, United Kingdom; ^2^Department of Small Animal Medicine and Surgery, University of Veterinary Medicine Hannover, Hanover, Germany; ^3^MRC Brain Network Dynamics Unit, Nuffield Department of Clinical Neurosciences, University of Oxford, Oxford, United Kingdom; ^4^Surrey Sleep Research Centre, University of Surrey, Guildford, United Kingdom; ^5^UK Dementia Research Institute, Care Research and Technology Centre, Imperial College London and The University of Surrey, Guildford, United Kingdom; ^6^Department of Neurology, Mayo Clinic, Rochester, MN, United States; ^7^Department of Clinical Neurophysiology, King's College Hospital NHS Trust, London, United Kingdom

**Keywords:** deep brain stimulation, centromedian thalamus, circadian, entrainment, epilepsy, chronotherapy, status epilepticus, Arnold tongues

## Abstract

Circadian and other physiological rhythms play a key role in both normal homeostasis and disease processes. Such is the case of circadian and infradian seizure patterns observed in epilepsy. However, these rhythms are not fully exploited in the design of active implantable medical devices. In this paper we explore a new implantable stimulator that implements chronotherapy as a feedforward input to supplement both open-loop and closed-loop methods. This integrated algorithm allows for stimulation to be adjusted to the ultradian, circadian and infradian patterns observed in patients through slowly-varying temporal adjustments of stimulation and algorithm sub-components, while also enabling adaption of stimulation based on immediate physiological needs such as a breakthrough seizure or change of posture. Embedded physiological sensors in the stimulator can be used to refine the baseline stimulation circadian pattern as a “digital zeitgeber,” i.e., a source of stimulus that entrains or synchronizes the subject's natural rhythms. This algorithmic approach is tested on a canine with severe drug-resistant idiopathic generalized epilepsy exhibiting a characteristic diurnal pattern correlated with sleep-wake cycles. Prior to implantation, the canine's cluster seizures evolved to status epilepticus (SE) and required emergency pharmacological intervention. The cranially-mounted system was fully-implanted bilaterally into the centromedian nucleus of the thalamus. Using combinations of time-based modulation, thalamocortical rhythm-specific tuning of frequency parameters as well as fast-adaptive modes based on activity, the canine experienced no further SE events post-implant as of the time of writing (7 months). Importantly, no significant cluster seizures have been observed either, allowing the reduction of rescue medication. The use of digitally-enabled chronotherapy as a feedforward signal to augment adaptive neurostimulators could prove a useful algorithmic method in conditions where sensitivity to temporal patterns are characteristics of the disease state, providing a novel mechanism for tailoring a more patient-specific therapy approach.

## Introduction

Physiological rhythms play a role in both normal homeostasis and disease processes, yet the design of active implantable medical devices often does not fully exploit them, especially in brain stimulators. For example, in the treatment of epilepsy with deep brain stimulation (DBS), the default stimulation approach is to apply high-frequency (HF) stimulation in an attempt to suppress seizure propagation (Fisher et al., 2010)—a method adapted from the successful treatment of Parkinson's disease (PD). While beneficial in many cases, occasionally resulting in periods with seizure freedom (Velasco et al., 2007; Valentín et al., 2013), an exploration of alternative strategies, or a combination of strategies (Schulze-Bonhage, 2019) could give new insights for epilepsy treatment. Similar opportunities exist in other disease states such as movement disorders and neuropsychiatry. One approach is to exploit the precise digital time control of implantable systems to interact with the rhythmic processes in the brain.

Normal and pathological rhythms arise at multiple timescales. At one temporal extreme, 24 h (circadian) and multiday (infradian) seizure patterns are observed in epilepsy (Baud et al., 2018; Gregg et al., 2020; Leguia et al., 2021). Despite these temporal fluctuations, current FDA-approved DBS and responsive neurostimulation (RNS) devices for control of seizures run a fixed algorithm regardless of the time. Vagal nerve stimulators do enable two settings for implementing diurnal control, which show promise for managing side-effects and correlating therapy with symptoms (Fisher et al., 2021). Similarly, disrupted sleep-wake cycles are a common co-morbidity of PD, depression and epilepsy, but current DBS devices default to fixed, tonic stimulation parameters that are configured based on an assessment of efficacy during a daytime follow-up (Malhotra, 2018). At the faster end of the spectrum, thalamocortical oscillations are signatures of both healthy and diseased brain states that fluctuate in intensity on the order of tens to hundreds of milliseconds (Oswal et al., 2013). While these oscillations are used for adaptive algorithms, the stimulation paradigm is still largely reliant on gating HF stimulation for suppressing these lower frequency oscillations (Little et al., 2013; Priori et al., 2013; Swann et al., 2018). Stimulation at lower frequencies, utilizing oscillation frequencies recorded during natural behavior, however may provide additional benefits over HF stimulation due to the entrainment properties of the target neural population. DBS parameters could in principle be tuned to act as a “digital chronotherapy” that modulates endogenous rhythmicity in brain activity over multiple timescales.

**Figure 1:**
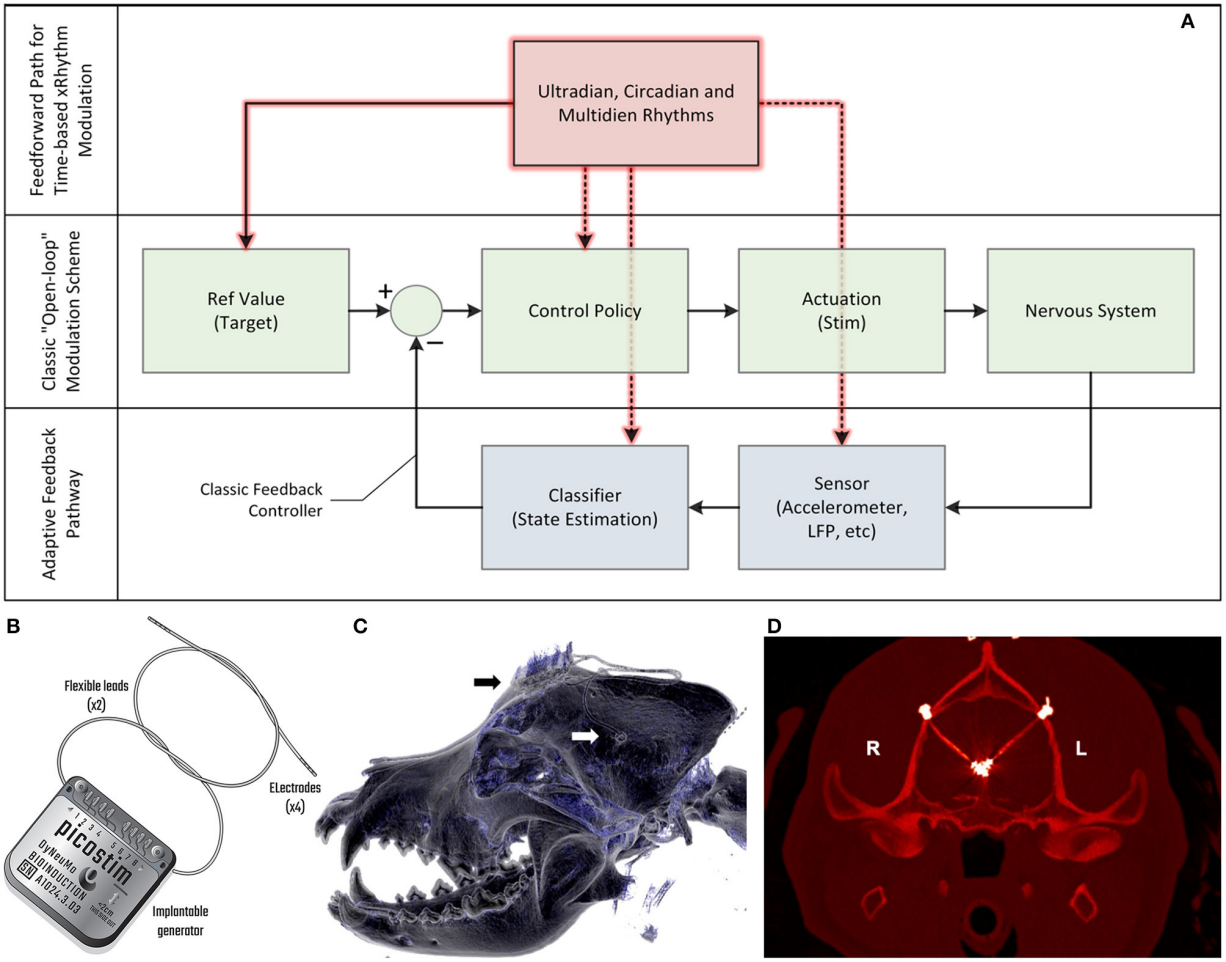
Algorithm model for therapy, device block diagram and postsurgical outcome. **(A)** The model for the control algorithm in the device. The typical “open loop” pathway is illustrated in green. A clinician generally captures a reference value and control policy based on experience and observation, setting a stimulation state for the device that interacts with the nervous system. A “closed loop” pathway, illustrated in blue, can be constructed by adding a sensor and classifier that can then adjust stimulation accordingly through a control policy; this adaptive feedback pathway allows for continuous refinement based on immediate patient state. A feedforward pathway, illustrated in red, can help to account for variability linked to set temporal rhythms, similar to circadian cycles observed in physiology control pathways. The feedforward pathway can adjust multiple parameters in the open and adaptive loops, including reference values for measured variables, how to stimulate, which sensors to use and the classification objectives. The combination of feedforward and feedback pathways aims to optimize predictive and responsive stimulation control. **(B)** The Picostim-DyNeuMo cranial mounted deep brain stimulator used in this study. For device capabilities refer to Toth et al. (2020). Note that the research tool is upgradeable through the firmware and software versions. **(C)** Postsurgical imaging shows a reconstructed 3D computed tomography (CT) scan of the canine's skull with the guide tube hub fixed in the drilled hole in the left parietal bone (white arrow) and the stimulator fixed on the frontal bone (black arrow). Wires coming from both guide tubes are connected with the stimulator. **(D)** Implanted guide tubes and wires in a transversal CT scan in the plane of the target structures. Note that the strong hyperintense signals around the target positions represent CT metal artifacts deriving from the electrode plates (*n* = 4, each) at the tip region of the wires. Please see supplemental methods for more details.

In this case study, we apply a new implantable stimulator in the centromedian nucleus of the thalamus (CMN) that implements multi-scale, rhythm-entrained stimulation as an experimental medicine treatment for SE. For human generalized seizures, the CMN is involved early or late in the seizure and when involved, appears to lead the cortex (Martín-López et al., 2017). Probably for this reason, this nucleus appears to be particularly useful for the treatment of super refractory SE in human patients (Valentín et al., 2012; Sa et al., 2019; Stavropoulos et al., 2021). SE is a serious ictal condition that is considered an emergent situation and can be fatal if these self-sustaining seizures cannot be interrupted. The stimulator's control algorithm applies feedforward input to supplement both open-loop and adaptive methods (Figure 1A). This integrated algorithm allows for electrical stimulation paradigms to be adjusted in response to slowly varying (e.g., diurnal/circadian) patterns through temporally-based adjustments of stimulation and algorithm sub-components, while also enabling adaptive stimulation based on immediate physiological needs such as a breakthrough seizure in epilepsy. The use of embedded field-potential sensing enabled subject-specific characterization of thalamocortical network activity. The field-potentials guided the application of targeted stimulation entrainment as an attempt to reinforce “beneficial” rhythms and avoid pathological ones. In aggregate, the physiological sensors and embedded timing control can serve to optimize the baseline stimulation circadian pattern as a digital zeitgeber, complementing or reinforcing existing zeitgebers.

## Case Overview

A 4-year-old, mixed-breed (Newfoundland/Saint Bernard), neutered male dog weighing 60 kg was presented with severe drug-resistant idiopathic epilepsy, at the Tier II confidence level of diagnostic certainty (De Risio et al., 2015). The carer's seizure diaries were used for comparative analysis of seizure type prevalence and frequency, seizure-free episodes and semiology of cluster seizures before and after surgery. The dog, treated as a veterinary patient, did not adequately respond to an array of antiseizure medication; treatment consisted of phenobarbital, potassium bromide, imepitoin, topiramate and gabapentin in various combinations (see **Figure 4** for details). Multiple dosages of diazepam or levetiracetam as pulse therapy were used following any given seizure to prevent cluster seizures (Packer et al., 2015). The dog's diet was enriched with 6% medium-chain triglycerides (MCT) with the goal to improve seizure control (Berk et al., 2020).

None of the epilepsy management options provided an adequate response and seizure severity increased to frequent SE. As no further medical treatment was available under the German Medicinal Products Act, the carer elected and gave informed consent for attempting DBS for epilepsy management. The Picostim-DyNeuMo research system (Bioinduction, Bristol, UK) was implanted with bilateral electrodes targeting the CMN, with the implantable pulse generator placed subcutaneously on the frontal cranium (Figure 1); refer to the supplemental methods for details. The Picostim-DyNeuMo can record intracranial signals and be remotely accessed for monitoring and therapy refinement; embedded circadian schedulers and sensors allow for adaptation of stimulation based on temporal patterns and inertial signals as well (Toth et al., 2020).

## Methods

Initially after implantation, HF stimulation was used for stimulation consistent with prior reports of CMN stimulation (130 Hz/90 μs). However, in the first post-implant cluster seizures, increasing HF amplitudes led to intolerable side-effects without seizure cessation (head-pulling and other involuntary motion) and the cluster sequence proceeded unabated. This motivated the use of an analytical approach for low frequency entrainment.

### Theoretical Mechanism for Parameter Selection: Arnold Tongue Analysis for Estimation of Entrainment

Our aim was to select stimulation frequencies which would reinforce neurotypical physiological behavior and avoid pathological rhythms. Prominent mesoscopic neural rhythms can be entrained through periodic electrical stimulation with specific amplitude and frequency predicted by Arnold tongues analysis. Entrainment may be subharmonic, characterized by a winding number *p:q*, with *p* and *q* integers, where *p* is the average number of oscillations achieved by the rhythm for a given *q* periodic pulses of the driving stimulation. Arnold tongues (Arnol'd, 1983; Pikovsky et al., 2002) can be observed in the stimulation frequency/amplitude space as patterns of constant winding number, typically elongated and triangular in shape. The *p:q* Arnold tongue represents the range of stimulation frequencies and amplitudes compatible with *p:q* entrainment. Arnold tongues have previously been reported in computational models of brain circuits, in particular in the context of circadian rhythms (Bordyugov et al., 2015; Skeldon et al., 2017) and transcranial stimulation (Trevisan et al., 2006; Ali et al., 2013; Herrmann et al., 2016).

**Figure 2:**
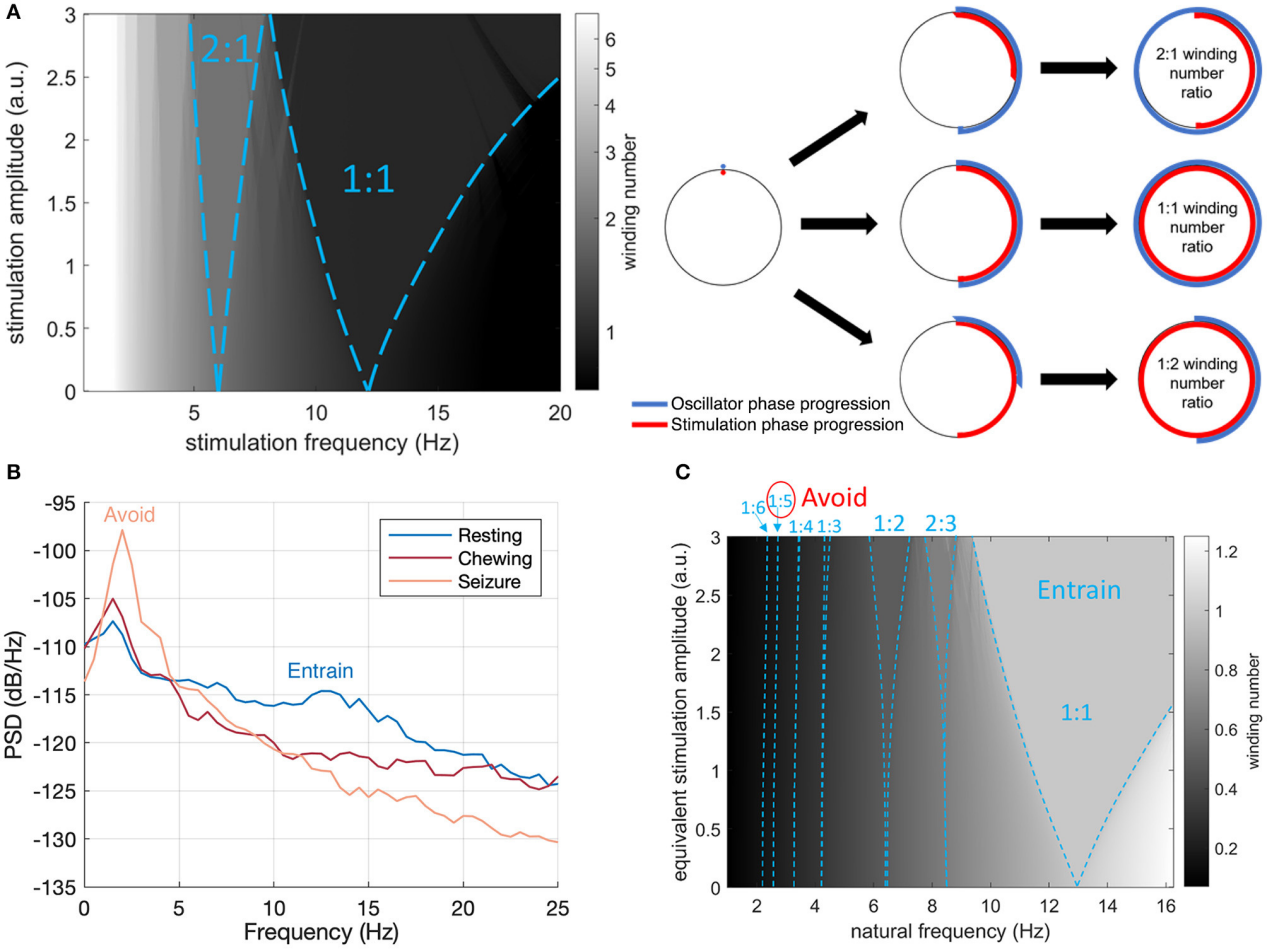
Arnold Tongues and Stimulation Frequency Strategy. **(A)** Winding number and Arnold tongues in the sine circle map as a function of stimulation (driver) frequency and amplitude for an oscillator with a natural frequency of 13 Hz. Arnold tongues correspond to areas of constant winding number. The 1:1 tongue (winding number of 1) and the 2:1 tongue (winding number of 2) are highlighted with blue dashed lines. For stimulation parameters falling within the p:q tongue, the rhythm will be entrained at p/q times the stimulation frequency. The sine circle map was simulated as $\theta_{i+1}=\;\theta_{i}+2\pi \left(\frac{f_{0}}{f_{s}}\right)+I\;\sin\theta_{i}$, where ***θ***_***i***_ is the oscillator phase right after stimulation pulse ***i***, ***f***_**0**_ is the natural frequency of the oscillator, ***f***_***s***_ is the stimulation frequency and ***I*** is the stimulation amplitude. The winding number was calculated after ***N*** = **50** stimulation pulses as the average of $\frac{\left(\theta_{N}-\theta_{0}\right)}{\left(2\pi N\right)}$ over 20 trials with random initial phases ***θ***_**0**_ uniformly distributed between **0** and **2π**. **(B)** Intracranial field potentials from the implanted signals (left hemisphere, contacts 0–3) remotely accessed through wireless telemetry. Representative signals were gathered during different activities of daily living to characterize frequency content. The stimulation therapy strategy aims to entrain the healthy rhythm around 13 Hz, while avoiding the peak at 2 Hz observed during seizure. **(C)** Illustration of the final stimulation strategy. The winding number in the sine circle map is shown here for a fixed stimulation frequency (13 Hz) as a function of natural frequency (e.g., inherent thalamocortical rhythm) and stimulation amplitude. Selected Arnold tongues are highlighted in blue. Stimulation at 13 Hz can reliably entrain the desired 12 Hz thalamocortical oscillation (large 1:1 tongue) while avoiding induction of pathological tongues in the region of 2–3 Hz. The 1:5 and 1:6 tongues obtained from 13 Hz stimulation are indeed so narrow that they will not lead to any entrainment in practice. To account for the fact that neural oscillations at lower frequencies typically have higher power (**1**/***f*** power law) and would therefore require more energy to entrain, the vertical axis represents equivalent stimulation amplitude (stimulation amplitude multiplied by ***f***_**0**_/***f***_**max**_, where ***f***_**0**_ is the natural frequency and ***f***_**max**_ the maximum frequency shown). This is conservative as the 1:5 and 1:6 tongues disappear at higher stimulation amplitudes.

The concept of Arnold tongues can be illustrated using the simplest model describing the influence of periodic stimulation on an oscillator. This model is the sine circle map (Glass and Mackey, 1979; Perez and Glass, 1982; Glass, 2001), where a phase oscillator with constant natural frequency is forced by periodic stimulation of controlled frequency and amplitude. A stimulation pulse will advance or delay a neuron's phase depending on where the neuron is in its firing cycle and on the neuron's type (Stiefel et al., 2008). Similarly, stimulation in the sine circle map advances or delays the phase of the oscillator, such that the change is proportional to the sine of the oscillator's phase at the time of stimulation. Varying stimulation frequency and amplitude reveals a family of Arnold tongues as shown in Figure 2 for a natural frequency of 13 Hz. Highlighted in Figure 2A are the 1:1 and 2:1 tongues, which encompass stimulation parameters resulting in the oscillator frequency being entrained at exactly the stimulation frequency and at twice the stimulation frequency, respectively. Since the 1:1 tongue is the largest, 1:1 entrainment is the easiest to achieve in practice. For a fixed stimulation frequency, a broader range of natural frequencies can follow 1:1 entrainment, which will therefore be most robust to perturbations acting to change the rhythm's natural frequency.

### Therapeutic Strategy—Basal Stimulation Frequency and Fast Adaptation

With remote telemetry, we were able to assess the spectral content of thalamocortical signals from our dataset based on prior characterization studies; representative power spectral density (PSD) plots are included in Figure 2B. Applying the entrainment hypothesis, we remotely tuned the stimulation frequency to the canine's dominant rhythm during restful, alert activity (13 Hz/350 μs/1.3 mA bilateral), while trying to avoid a sub-harmonic rhythm which might align with the 2 Hz oscillation that correlated with seizure onset and initiation. Similar low frequency rhythms have also been suggested to induce absence seizures in human subjects (Velasco et al., 1997). The final entrainment model that guided therapy is summarized in the Arnold Tongue plot of Figure 2C. The adoption of this setting coincided with the end of the immediate cluster seizure event and it has been used thereafter as the default stimulation pattern. As an emergency fall-back for breakthrough seizures, a HF mode with elevated amplitude (130 Hz/90 μs/1.5 mA bilateral) was implemented which could be triggered by the carer through tap activation, using the built-in accelerometer (Figure 1B). Note that the levels for the emergency HF stimulation would not be tolerated during normal activities of daily living, e.g., it can induce reversible head-pulling, but were acceptable for an emergent state.

### Therapeutic Strategy—Diurnal Rhythms and Slow Adaptation

Since physiological rhythms can vary throughout the day (Gregg et al., 2020; Leguia et al., 2021), the stimulation might benefit from temporal adjustments regardless of immediate physiological state. In case of our canine stimulation could lead to hypervigilance, so the therapy was adjusted to vary over time, supplemented by adaptive transitions based on activity/inactivity.

**Figure 3:**
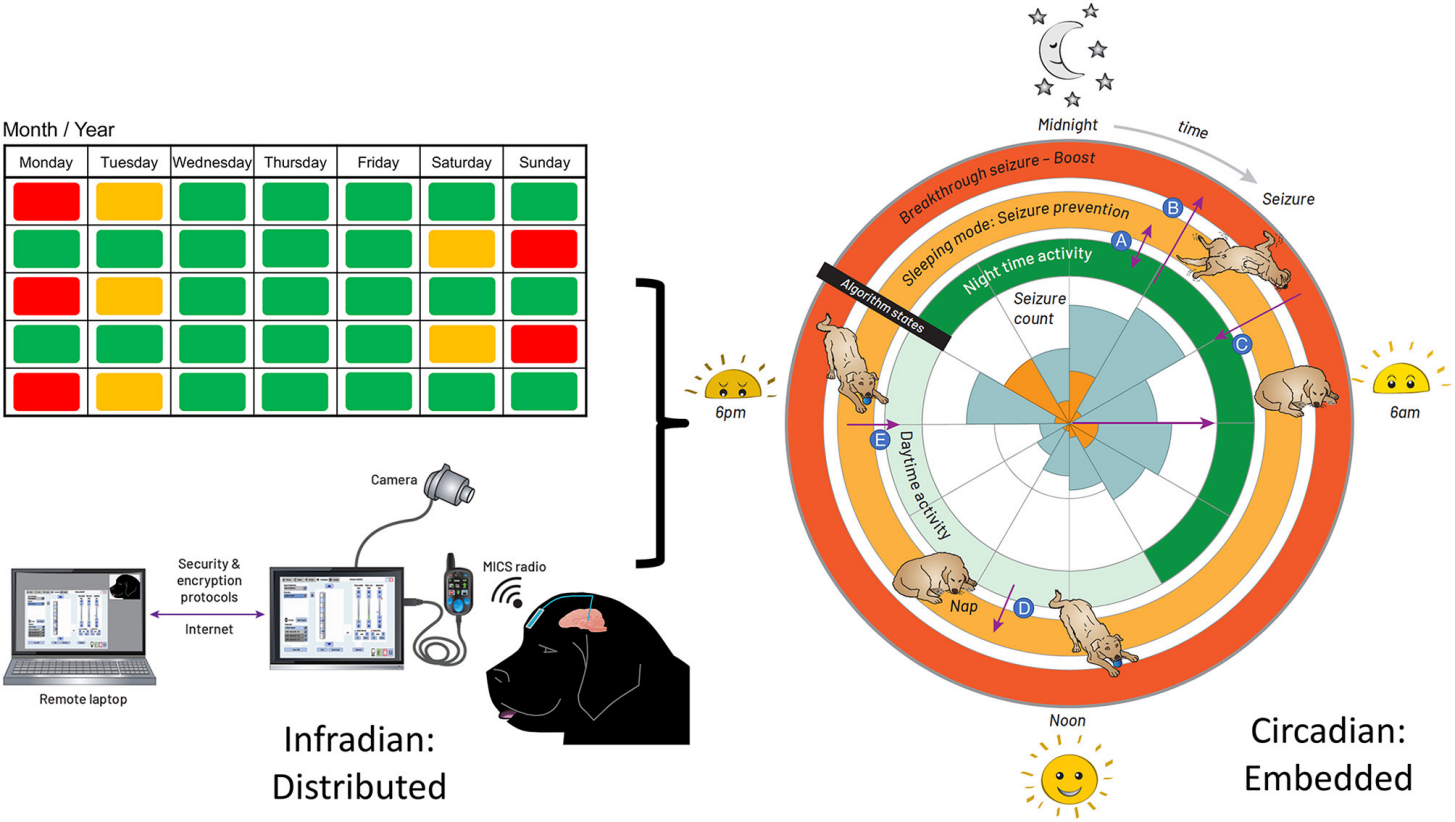
Integrated Circadian-Adaptive Mode Algorithm Strategy. Illustration of the algorithm timing strategy. **Left:** extended time (e.g., 2-week infradian) updates are provided over an encrypted internet link with a local password-protected application running on a surface tablet via the patient controller. The USB connector between the patient programmer and tablet is for in-clinic programming. Research subjects use the handheld controller for at-home recharge and manual adjustments. **Right:** the embedded algorithm illustrated with a rose plot. The inner circle represents the seizure count from the diary, kept by the carer, up to the date of DBS activation; the orange tiling is the timing of first seizure onset, while the blue account for all seizures in a cluster. The inner ring of stimulation is the *default state* when the dog is active. When the accelerometer detects an inactive state for 4 min, the algorithm transitions to the middle ring of stimulation for the *sleeping mode*. The outer ring is the *boost mode* for breakthrough seizures. (A) going in and out of sleep during the night, while in night-time mode, the stimulation switches from night-time to the sleeping mode and *vice versa*. (B) suffering a seizure during the default mode, the carer taps on the site of the implant to trigger the boost mode. (C) the stimulation returns from boost mode to the default mode within 30 min. (D) taking a nap during the day, the stimulation switches from the default mode to the sleeping mode. (E) waking up from the nap, the stimulation goes from sleeping mode to default mode within 30 min.

The temporal pattern to stimulation adjustment was introduced so as to align maximum stimulation intensity with times of peak seizure activity, as recorded in the seizure diary kept by the carer. The historic seizure activity up to the date of implantation is presented in a rose plot, inset in the right panel of Figure 3. The timing of seizures motivated a circadian adaptive pattern for stimulation; note that seizures were generally linked to sleep states according to the carer.

The aim was to account for immediate variation in activity, while accounting for daytime naps, since the highest probability of seizures correlated with the sleep state. The final adaptive algorithm, merging chronotherapy and sensor-based inputs, consisted of three layers of control with increasing stimulation intensity: (1) a circadian basal rate while the dog is awake and active; (2) a protective sleep mode with elevated entrainment stimulation; and (3) a high-amplitude, HF stimulation pattern to try and abort a breakthrough seizure through existing DBS methods. The embedded algorithm is illustrated by the circles enclosing the rose diagram in Figure 3. The inner ring of stimulation is the default state at 13 Hz when the dog is active; the *night-time* activity uses elevated stimulation 0.7 mA for additional protection, while the *daytime* stimulation is lowered to 0.5 mA to avoid any side-effects of stimulation and conserve energy during low seizure probability intervals. When the accelerometer detects an inactive state for 4 mins, the algorithm transitions to the middle ring of stimulation for the *sleeping mode*, which elevates stimulation amplitude to 1.3 mA at 13 Hz to provide greater entrainment during the increased risk of seizures during sleep. Finally, the outer ring, or *boost mode*, is designed for breakthrough seizures, activated by the carer with a single tap on the device programmed with a detection threshold of 7 g in the z-axis (orthogonal from the device plane). In this mode, a burst of 130 Hz, 1.5 mA bipolar stimulation is provided to interrupt a sustained seizure.

We remotely synchronized the device for the longer infradian rhythms (Baud et al., 2018). Remote telemetric access allowed us to characterize physiology and reprogram the system in the home environment, as well as check battery levels and tissue-electrode interface impedances. On the left side of Figure 3, extended time (e.g., 2-week infradian) updates are provided over an encrypted internet link with a local password-protected application running on a surface tablet. The patient controller is used to wirelessly update the embedded stimulation parameters.

## Results

### Prevention of Status Epilepticus

**Figure 4:**
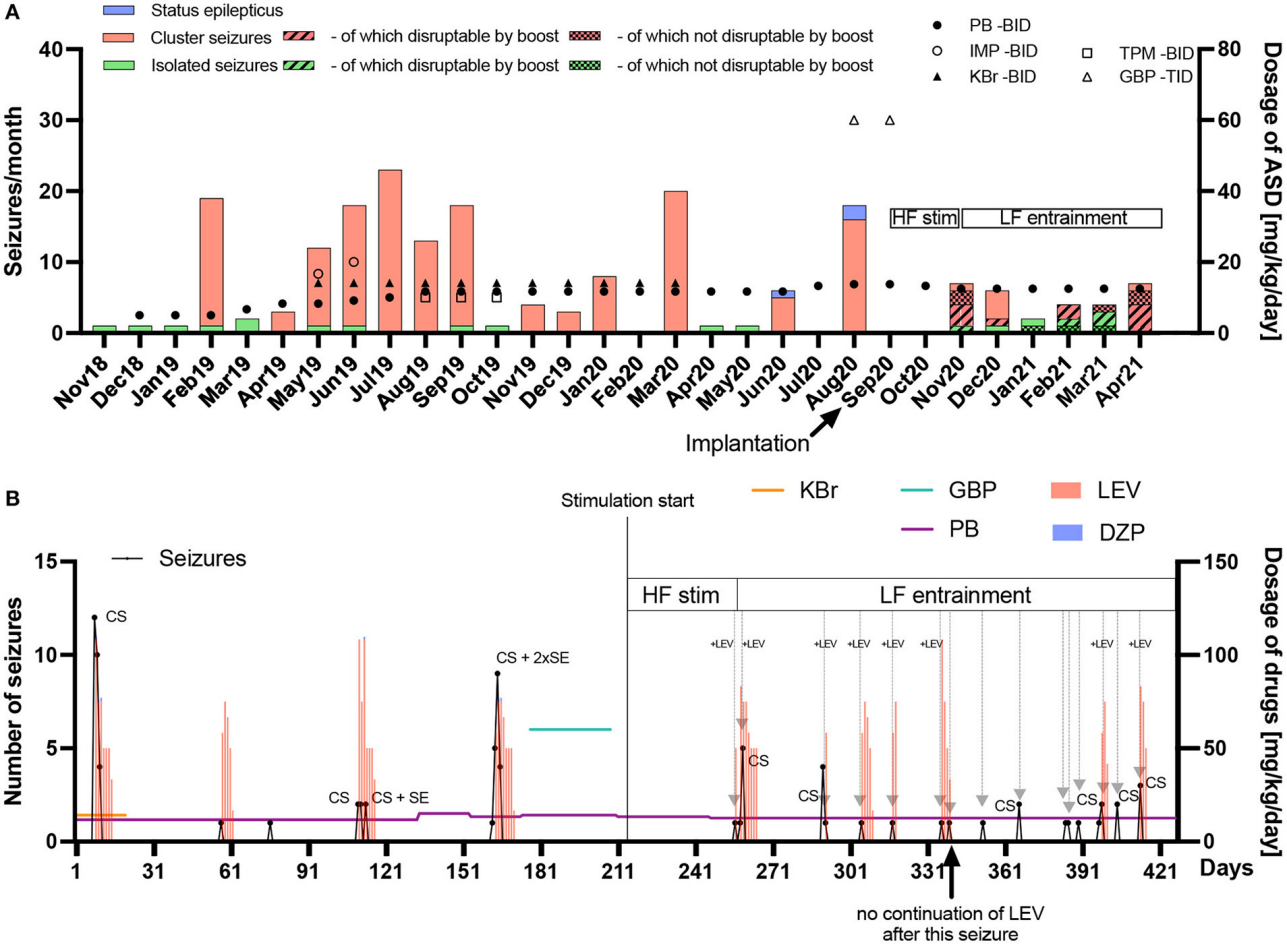
Frequency and chronic/acute pharmacological treatment of seizures until seven months after implantation. **(A)** The seizure frequency (left y-axis) and average daily antiseizure drug (ASD) dosage in mg/kg/day (right y-axis) of phenobarbital (PB), imepitoin (IMP), potassium bromide (KBr), topiramate (TPM) and gabapentin (GBP) twice (BID) or thrice (TID) daily are shown for every month since epilepsy onset in November 2018. When stimulation was started, no further status epilepticus (SE) occurred. After implantation, high frequency (HF) stimulation was applied after seizures occurred in order to prevent SE or further cluster seizure evolution. Since HF did not bring the desired result in the first cluster seizure (November 2020), the frequency was subsequently changed to low frequency (LF) entrainment adapted to the canine's local field potentials. PB was chronically administered as monotherapy (reduction from 13.3 to 12.5 mg/kg/day in November 2020), while other chronic medical and dietary therapy with medium-chain triglycerides was stopped after surgery. With the HF bursting *boost mode* for the interruption of ongoing seizures four seizures out of six attempts in November 2020 were interrupted. One seizure was noticed too late. In December 2020, one seizure was interrupted while five further ones occurred without interruption attempt. In January 2021, one attempt of seizure interruption was without success, while another seizure was noticed too late. In February 2021, two out of three attempts of seizure interruption were successful. In March 2021, three seizures were interrupted while two continued after attempt. In April 2021, four seizures were interrupted, two continued and one was noticed too late. The overall success rate of interruption attempts was 64%. Electrical stimulation has the potential of reducing acute and chronic pharmacological interventions. **(B)** The seizure number (left y-axis) in black dots and lines and average daily ASD dosage in mg/kg/day (right y-axis) for chronic (horizontal colored lines) and emergency treatment (vertical bars) with levetiracetam (LEV) or diazepam (DZP) are shown on a day-to-day basis since begin of March 2020 until end of April 2021 (seven months pre and post implantation). Gray arrows represent successful avoidance of further seizure occurrence/evolution after a single seizure or in cluster seizures (CS) via stimulation with (+LEV) or without LEV intervention. It turned out that initially, only the combination of acute LEV treatment and LF-entrainment prevented or interrupted cluster seizure evolution. Since February 2021, however, LEV was not administered after seizure occurrence due to severe side-effects, with LF-entrainment alone achieving cluster cessation, except in two CS events which consisted of rapid successive but few seizures in April 2021. Eight seizure evolution interruptions were conducted together with LEV and seven without LEV after stimulation onset, with only seven interruptions with LEV during LF-entrainment. DBS has the potential of reducing acute pharmacological interventions.

The data is summarized in Figure 4 based on seizure diary and care plan summary. Figure 4A shows the seizure number and medication dosage per month since epilepsy onset, while Figure 4B shows the same data on a daily basis from 7 months before until 7 months after implantation and stimulation onset.

Status epilepticus: 3 months before implantation, the seizure severity increased dramatically. Seizures regularly escalated into SE, with three occurring prior to surgery, requiring the use of rescue intervention. After implantation and the use of described stimulation patterns, no SE occurred.

Rescue medications: After implantation of the device, levetiracetam administration as pulse therapy, with major side-effects, was initially continued after seizure occurrence in order to further interrupt cluster seizure evolution or SE (repetitive administration every 8 h with successive dose reduction over several days). It was possible to successfully break the cluster seizure emergence or evolution in seven seizure occurrence periods (total of nine seizures) via stimulation only without administering levetiracetam as additional rescue medication in these periods (Figure 4B). Phenobarbital as chronic treatment was continued over the whole observation time after implantation with a dose reduction from 13.3 to 12.5 mg/kg/day in November 2020. The carer also stopped MCT supplementation and the other antiseizure medications.

Breakthrough seizure intervention: In terms of proactively interrupting ongoing seizures by the carer, the *boost* (HF burst) emergency mode disrupted 14 seizures, while eight seizures continued after interruption attempt. Another eight seizures were not interrupted because they were noticed too late or the *boost mode* was deactivated at those time points. The success rate of the active interruption attempts was thus approximately 64%.

### Significant Trends for Reduction of Cluster Seizures

General trends: The mean number of seizures during a seizure occurrence period, i.e., periods of isolated seizures (IS) or coherent cluster seizures (CS), as well as the mean duration of these periods (IS = 0 h; CS > 0 h) as a measurement for severity were assessed before and after start of low frequency (LF) entrainment (including preoperative seizures). Since all SE were part of a CS event, they were included for these measurements. The overall number of seizures within a seizure occurrence period since epilepsy onset was 4.67 ± 5.99 [mean ± SD, range 1–26] with seizures occurring over a time period of 16.21 ± 21.35 h [mean ± SD, range 0–74.5] per seizure occurrence period. Before the start of LF entrainment (including preoperative seizures), the number of seizures during an ictal period was 5.84 ± 6.73 [mean ± SD, range 1–26] vs. 1.77 ± 1.24 [mean ± SD, range 1–5] after LF entrainment started [*p* < 0.05]. The time between the first and last seizure during a seizure occurrence period was 20.57 ± 23.42 h [mean ± SD, range 0–74.5] vs. 5.48 ± 8.95 h [mean ± SD, range 0–24] before and after start of LF entrainment (including preoperative seizures), respectively [*p* < 0.05].

The graph in Figure 4A shows that the seizure number decreased in general without showing increased episode frequency, while the graph in Figure 4B shows that seizure episodes got more frequent, but less severe than before stimulation.

## Discussion

Physiology generally merges feedforward (e.g., circadian) and feedback (e.g., homeostatic) control mechanisms. Implantable bioelectronic systems, while capable of precision timing and adaptive control, have not yet fully adopted a similar integrated control scheme. One reason is the complexity of additional control variables that might burden the clinician while configuring the system; ultimately an additional benefit must be demonstrated to justify the added complexity. However, many systems might yield immediate benefit by simply synchronizing stimulation modification to other diurnal variables such as medication timing. For example, fixed tonic stimulation of neural targets that couple into the reticular activating network have shown impact on sleep architecture (Voges et al., 2015). The fact that many areas of neuromodulation—epilepsy, PD, chronic pain and depression—have sleep co-morbidities also motivates an exploration of aligning stimulation with diurnal cycles to both enhance therapy and avoid side-effects (Sladky et al., 2021).

Alignment of rhythms at multiple scales requires a consideration of entrainment properties. We used the model of Arnold tongues from dynamic systems theory for selecting objectively the stimulation frequency. Arnold tongues can be useful for considering how stimulation might lead to non-linear effects which might not be intuitively predicted and have surprising side-effects. For example, PD patients can have half-harmonic locking of gamma rhythms (e.g., 65 Hz peak) in response to 130 Hz stimulation frequency (Swann et al., 2016). This non-linear mapping of brain stimulation to network oscillations might result inadvertently in reinforcing undesirable side-effects such as dyskinesia (Swann et al., 2016). Critically, these observations support the hypothesis that the conditions for Arnold tongues and subharmonic entrainment of the cortex are present with DBS of the basal ganglia. There is evidence that the alpha rhythm might also play a role in epilepsy (Abela et al., 2019); our strategy of attempting to entrain at a slightly higher frequency might also provide potential benefits, which warrants further investigation. For longer temporal scales such as circadian rhythms, the impact of stimulation as a “digital zeitgeber” might also result in additional phase shifts between existing zeitgebers (e.g., daylight or eating) and the endogenous circadian rhythm. Such phase shifts could either help restore sleep patterns, or create undesirable side effects, depending on the entrainment characteristics. Validating and applying these non-linear models of entrainment with additional clinical research might help to optimize the timing of stimulation at multiple temporal scales of physiology.

Finally, risk mitigations for novel adaptive stimulation methods must be considered. In the Picostim-DyNeuMo system, these mitigations include constraining the stimulation space to a predefined set of parameters screened by clinicians. In addition, we define a fallback program that a patient or carer can revert to in the case of issues arising with the adaptive mode. This action resets the system to open-loop stimulation, which is the default for most existing neuromodulation approach. An overview of the risk strategy method can be found in Gunduz et al. (2019).

## Case Limitations

This case report has several limitations. The study is of a single canine, which limits the statistical conclusions, but primarily serves as pilot validation of the implant technology. Although our results are consistent with recent human case studies (Valentín et al., 2012; Sa et al., 2019; Stavropoulos et al., 2021) where the benefit of CMN thalamic stimulation at LF relative to HF was observed providing further support for the clinical value of thalamic LF stimulation, additional tests are needed. We adapted the stimulation based on physiological measurements and chose a higher frequency stimulation for entrainment. In addition, the application of experimental medicine prevented us from applying a self-control such as terminating treatment and assessing the impact on seizures. During the course of stimulation exploration, however, we were able to confirm that stimulation at 2 Hz in the CMN increased the probability of seizures (induction <24 h after setting) consistent with previous observations (Velasco et al., 1997). The case is ongoing and the reported results are limited to the first 7 months of follow-up. Prior studies of epilepsy have shown changing efficacy over many years, although arguably for the better on average (Nair et al., 2020). In addition, we are relying on manual seizure diary which can be unreliable (Ukai et al., 2021); the strongest evidence we have are the SE events, which are severe enough to not be missed by the carer. Finally, the Picostim-DyNeuMo is limited by law to investigational device applications at this time.

## Summary

The synchronization of brain stimulation to endogenous rhythms is an emerging concept for therapy optimization. The use of digitally-enabled chronotherapy as a feedforward signal to augment adaptive neurostimulators could prove to be a useful algorithmic method where sensitivity to temporal rhythms are characteristics of the disease state, tailoring a more patient-specific therapy approach. Computational models predicting Arnold tongues can also guide the design of patient-specific stimulation parameters, which has often been a heuristic process. In this proof-of-concept study, using a novel chronotherapy-enabled device in a canine with severe drug-resistant idiopathic epilepsy, these methods had favorable outcomes in terms of improving seizure semiology, reducing coherent cluster seizures and controlling (or avoiding) SE. The carer reports a reduced fear of seizures and improved personal quality of life based on the reduction of seizure severity with the stimulation. The adaptability of this approach allows for individualized therapies that are supported by emerging adaptive devices with both physiological sensing and chronotherapy capability. In addition, this report of DBS in canine epilepsy further highlights the possibility of using veterinary medicine as a vehicle to test new device and treatment paradigms (Potschka et al., 2013).

## Data Availability Statement

The raw data supporting the conclusions of this article will be made available by the authors, without undue reservation.

## Author Contributions

TD and HV: conceptualization. TD, HV, and D-JD: design of the study. SM, HV, and FK: surgery and veterinary care. TD, D-JD, GW, and AV: algorithm definition. MZ and MB: algorithm implementation. SM, MZ, RT, RB, BD, and JS: data analysis. TD, SM, FK, RT, MZ, BD, and JS: figures. TD, MZ, SM, and HV: writing original draft. All authors writing, reviewing and editing.

## Funding

MZ and JS were supported by the Medical Research Council grant MC_UU_00003/3, RB and BD by grants MC_UU_12024/5 and MC_UU_00003/1. TD was funded by the Royal Academy of Engineering.

## Conflict of Interest

TD has business relationships with Bioinduction for research tool design and deployment. GW has a financial interest in Cadence Neuroscience Inc. The remaining authors declare that the research was conducted in the absence of any commercial or financial relationships that could be construed as a potential conflict of interest.

## Publisher's Note

All claims expressed in this article are solely those of the authors and do not necessarily represent those of their affiliated organizations, or those of the publisher, the editors and the reviewers. Any product that may be evaluated in this article, or claim that may be made by its manufacturer, is not guaranteed or endorsed by the publisher.
