# Supplementary material for: Case Report: Embedding “Digital Chronotherapy” Into Medical Devices—A Canine Validation for Controlling Status Epilepticus Through Multi-Scale Rhythmic Brain Stimulation
Source: Front Neurosci. 2021 Sep 24;15:734265. doi: 10.3389/fnins.2021.734265 (PMC8498587; doi:10.3389/fnins.2021.734265)
Supplement: Supplementary file 1 [file Data_Sheet_1.PDF]

Case Report: Embedding "Digital Chronotherapy" into Medical Devices – A canine validation for controlling status epilepticus through multi-scale rhythmic brain stimulation

Supplementary Material

Mayela Zamora, Sebastian Meller, Filip Kajin, James J Sermon, Robert Toth, Moaad Benjaber, Derk-Jan Dijk, Rafal Bogacz, Gregory A Worrell, Antonio Valentin, Benoit Duchet, Holger A Volk, Timothy Denison

1 The Device

The Picostim-DyNeuMo system (Bioinduction, Bristol, UK), serves as a 7cc cranially-mounted brain pacemaker, supplemented with on-demand telemetric monitoring of neurobiological signals and flexible and remotely adjustment of stimulation parameters to the patient's individual needs. Details of the device are provided in (Toth et al. 2020). Feedback responses enabled by biological inputs from the built-in accelerometers and from telemetric measurements of neuronal signals together with high resolution temporal adjustments to stimulation parameters represent key features. Furthermore, feedforward paths can be configured by e.g. using clock and activity measurement, or manipulations, via accelerometry. This provides a highly flexible system which offers an investigational hub for neuroscience and for the development of new open and closed loop deep brain stimulation (DBS) solutions.

2 Presurgical Planning

Both centromedian nuclei (CMN) of the thalamus were chosen as targets, as its electrical stimulation was shown to be effective in people with refractory generalized epilepsy syndromes (Velasco et al. 2002; Valentín et al. 2013; Cukiert and Lehtimäki 2017). The StrykerNAV3® hardware and Stryker CranialMap® 3.0 software (Stryker Corporation, Kalamazoo, MI, USA) were used to perform presurgical planning and neuronavigation during surgery with related patient- and instrument-tracking devices. Magnetic resonance imaging and computed tomography (CT) images of the canine patient's skull and brain were merged for surgical planning of the bilateral tracks for the implantable Renishaw® neuroguide™ DBS electrode guide tubes (Renishaw plc., Gloucestershire, UK) and electrode placement. Bilateral trajectories (36mm each from the skull surface) were planned through the ectosylvian gyrus of the temporal lobe from a rostro-dorso-lateral to caudo-ventro-medial direction with entry points in the rostral portion of the parietal bones and passing through and ending slightly ventromedially from the CMN of the corresponding hemisphere in order to ensure electrode contact over the entire structure (Supplementary Figure 1).

Correspondence: mayela.zamora@eng.ox.ac.uk, sebastian.meller@tiho-hannover.de

3 Surgery

The veterinary department's routine protocol for peri- and postoperative anaesthesia and analgesia management for neurosurgery was used. A median incision from the caudal part of the nasal bones to the external occipital protuberance was performed and the temporal muscles were dissected medially in order to expose the parietal bones. A patient tracker for neuronavigation was mounted on the skull over the nasion. A hole (∅ 5mm) was drilled into the parietal bone on both sides, each at the previously defined entry points in the direction of the planned track and the dura mater was incised. Both guide tubes were shortened to 27mm since a tip clearance distance of 9mm was needed for the adequate delivery of the four electrode contacts of the implantable Bioinduction picostim DyNeuMo-1 25cm DBS Leads (Bioinduction, Bristol, UK) to the CMN. Guide tubes were mounted from distal on the Renishaw® neuroguide™ instrument guide rod (Renishaw plc., Gloucestershire, UK), which had been fixed in the Scopis Biopsy Guide (Scopis GmbH, Berlin, Germany) and tracked for neuronavigation. A plastic slider was fixed on the rod immediately above the guide tube hub in such a way that the distal ends of both the tube and the rod formed a flush closure. The slider prevented displacement of the tube and a too deep advance of the rod into the brain during lowering of the assembly. Once the rod with the guide tube inside the biopsy guide was exactly aligned above the drill holes thanks to neuronavigation, the rod was lowered along the planned track into the brain parenchyma until the protruding edge of the guide tube hub met the surface of the bone and the rod was removed from the brain. Neuronavigation allowed for continuous supervision of the correct lowering of the guide rod. The section of the leads to be implanted was marked with a suture and inserted into the guide tubes. Leads and guide tube hub were fixed to the bone with bone cement and both proximal ends of the leads were inserted into the Bioinduction picostim DyNeuMo-1 DBS implanted pulse generator (Bioinduction, Bristol, UK), which was attached to the frontal cranium with bone cement in a rubber sheath and fixed to the surrounding fascia with sutures. The muscle layer was sutured together over the stimulator and a routine closure was applied. Immediately after surgery CT imaging of the skull was performed in order to verify and compare the actual position of the guide tubes and leads with the pre-planned tracks.

4 Statistical Analysis

Where appropriate, Mann Whitney U test was used in order to compare seizure properties between the time periods before and after stimulation approach. Statistical analyses were performed with GraphPad Prism 9 (La Jolla, CA, USA). One-tailed tests were used and a $p \leq 0.05$ was considered significant.

Supplementary Figure 1: Presurgical planning

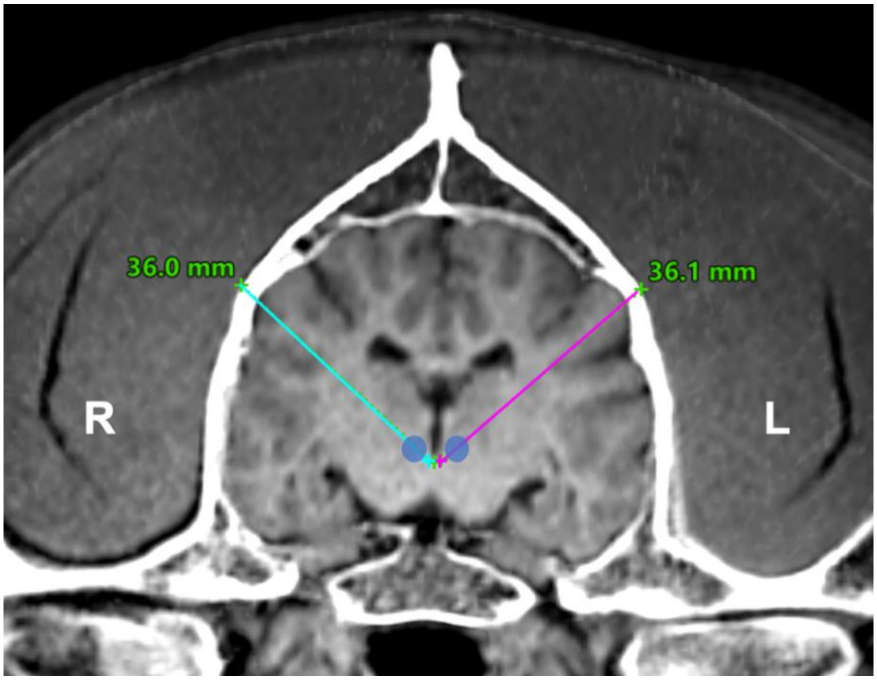

Presurgical planning with transversal merged magnetic resonance imaging and computed tomography scans of the skull and brain with the Stryker CranialMap® software. The blue (right hemisphere) and magenta (left hemisphere) trajectories represent the planned implantation sites and orientations of both guide tubes with the entrance points at the skull surface and the end points slightly ventromedially from the centromedian nuclei (CMN). The lengths of the planned trajectories are shown in green. Since a tip clearance of 9mm is needed for appropriate delivery of electrical information from the electrodes, guide tubes were shortened to 27mm each. Blue circles represent the CMN in both hemispheres.

5 References

Cukiert, Arthur and Kai Lehtimäki. 2017. 'Deep brain stimulation targeting in refractory epilepsy', *Epilepsia*, 58: 80-84.

Toth, R., M. Zamora, J. Ottaway, T. Gillbe, S. Martin, M. Benjaber, G. Lamb, T. Noone, B. Taylor, A. Deli, V. Kremen, G. Worrell, T. G. Constandinou, I. G. S. De Wachter, C. Knowles, A. Sharott, A. Valentin, A. L. Green and T. Denison. 2020. "DyNeuMo Mk-2: An Investigational Circadian-Locked Neuromodulator with Responsive Stimulation for Applied Chronobiology." In *2020 IEEE International Conference on Systems, Man, and Cybernetics (SMC)*, 3433-40.

Valentín, Antonio, Eduardo García Navarrete, Ramesh Chelvarajah, Cristina Torres, Marta Navas, Lelia Vico, Nerea Torres, Jesus Pastor, Richard Selway, Rafael G. Sola and Gonzalo Alarcon. 2013. 'Deep brain stimulation of the centromedian thalamic nucleus for the treatment of generalized and frontal epilepsies', *Epilepsia*, 54: 1823-33.

Velasco, Francisco, Marcos Velasco, Fiacro Jimenez, Ana Luisa Velasco, Beatriz Rojas and Martha Luisa Perez. 2002. 'Centromedian nucleus stimulation for epilepsy: Clinical, electroencephalographic, and behavioral observations', *Thalamus & Related Systems*, 1: 387-98.
